# Emotion-Related Visual Mismatch Responses in Schizophrenia: Impairments and Correlations with Emotion Recognition

**DOI:** 10.1371/journal.pone.0075444

**Published:** 2013-10-07

**Authors:** Gábor Csukly, Gábor Stefanics, Sarolta Komlósi, István Czigler, Pál Czobor

**Affiliations:** 1 Department of Psychiatry and Psychotherapy, Semmelweis University, Budapest, Hungary; 2 Translational Neuromodeling Unit (TNU), Institute for Biomedical Engineering, University of Zurich and ETH Zurich, Zurich, Switzerland; 3 Laboratory for Social and Neural Systems Research, Department of Economics, University of Zurich, Zurich, Switzerland; 4 Institute of Cognitive Neuroscience and Psychology, Research Center for Natural Sciences, Hungarian Academy of Sciences, Budapest, Hungary; 5 Nathan Kline Institute for Psychiatric Research, Orangeburg, New York, United States of America; University of Bern, Switzerland

## Abstract

**Background and Objectives:**

Mismatch negativity (MMN) is an event-related potential (ERP) measure of preattentional sensory processing. While deficits in the auditory MMN are robust electrophysiological findings in schizophrenia, little is known about visual mismatch response and its association with social cognitive functions such as emotion recognition in schizophrenia. Our aim was to study the potential deficit in the visual mismatch response to unexpected facial emotions in schizophrenia and its association with emotion recognition impairments, and to localize the sources of the mismatch signals.

**Experimental Design:**

The sample comprised 24 patients with schizophrenia and 24 healthy control subjects. Controls were matched individually to patients by gender, age, and education. ERPs were recorded using a high-density 128-channel BioSemi amplifier. Mismatch responses to happy and fearful faces were determined in 2 time windows over six regions of interest (ROIs). Emotion recognition performance and its association with the mismatch response were also investigated.

**Principal Observations:**

Mismatch signals to both emotional conditions were significantly attenuated in patients compared to controls in central and temporal ROIs. Controls recognized emotions significantly better than patients. The association between overall emotion recognition performance and mismatch response to the happy condition was significant in the 250–360 ms time window in the central ROI. The estimated sources of the mismatch responses for both emotional conditions were localized in frontal regions, where patients showed significantly lower activity.

**Conclusions:**

Impaired generation of mismatch signals indicate insufficient automatic processing of emotions in patients with schizophrenia, which correlates strongly with decreased emotion recognition.

## Introduction

Perception of emotional facial expressions has been shown to be closely related to psychosocial functioning and quality of life in schizophrenia [1]. An extensive body of research has accumulated suggesting a robust impairment in emotion recognition in schizophrenia, especially regarding facial emotion recognition [2].

While the behavioral indices of facial emotion recognition deficits in schizophrenia are robust, the underlying neurophysiological processes are still largely unknown. Although a large number of studies have investigated the electrophysiological correlates of conscious emotional face processing (see [3] for review), only a few studies investigated the automatic processing of unattended expressions, usually with healthy subjects [4], [5].

In the present study we investigated automatic change detection in facial expressions via the visual mismatch (vMM) component of the event-related potentials. vMM response is the visual counterpart of the auditory mismatch negativity (MMN: for review see [6]). The auditory MMN has been widely studied in schizophrenia, and reports usually indicate impaired automatic auditory processing [7]. Both the auditory MMN and vMM signals are typically elicited by stimuli with an infrequent (deviant) stimulus feature embedded in a stream of frequent (standard) stimuli. vMM response is elicited by deviant color [8], orientation [9], movement [10], spatial frequency [11], contrast [12], and even abstract sequential regularities of visual stimulation [13], see [14]–[16] for reviews). Mismatch responses are considered as automatic prediction error signals [17] representing the updating of generative models of environmental regularities after the violation of the model’s prediction by a deviant stimulus [18]. Urban et al found that deviant stimulus features (motion direction) elicited reduced vMM signal in schizophrenic patients [19].

The ERP paradigm applied in our study does not require overt responses to the face stimuli, allowing us to study the automatic processing of facial emotions presented outside of the focus of visual attention. Regarding its ecological validity, in real-life situations our attention is mostly engaged by events appearing in the center of the visual field, while important events (such as emotionally relevant stimuli) may emerge at the periphery. Furthermore, behavioral priming studies confirmed that affective processing occurs outside of the focus of visual attention [20]–[22].

Several studies demonstrated that vMMN is elicited by simple deviant features (see Kimura et al. [5] for a review, and Maekawa et al. [23] for a clinically-focused review ). To date only a few studies investigated visual mismatch negativity in healthy subjects using abstract regularities [24] or complex natural visual stimuli such as emotional facial expressions [5], [25], or body parts [13]. A recent study by Kimura et al. [5] reported that occipital, temporal and frontal regions play a major role in the generation of the facial expression-related mismatch response. As Stefanics et al. [25] summarized, occipital and temporal visual areas together with frontal generators automatically represent regularities in the emotional content of unattended faces appearing outside of the focus of attention and store them as predictive memory representations. The biological significance of such representation might be orienting our attention to sudden changes in emotional expression of conspecifics in our environment, analogously to auditory MMN [26], and also maintaining a predictive model of the environment. Although the processing of unattended facial emotions is likely to play an important role in social interactions, to our knowledge no study so far investigated the neural correlates of these processes in patients with schizophrenia.

We studied the differences between patients and control subjects by comparing their vMM responses to unattended rare (deviant) facial emotions embedded in a stream of faces expressing frequent (standard) emotions. We hypothesized that the vMM signal might be a sensitive indicator of compromised automatic information processing of emotional expressions in schizophrenia. Emotion recognition performance was evaluated in a separate behavioral test. To establish an association between the automatic vMM response and emotion recognition performance, we studied the correlation between behavioral performance and mismatch signal amplitudes. Based on well-known deficits in emotion processing in schizophrenia [2] we expected lower emotion recognition performance in patients. In conjunction with this, we also expected a significantly decreased mismatch response to emotional facial stimuli in patients with schizophrenia compared to controls. Finally, we hypothesized that the neural generators of the vMM response are located in occipital-temporal and frontal-prefrontal areas [5], and that the activity of these regions is decreased in schizophrenia.

## Materials and Methods

### Ethics Statement

The experiments were conducted in full compliance with the Helsinki Declaration and all relevant national and international ethical guidelines. The research was approved by the review board of the Semmelweis University, Budapest, Hungary. All procedures were carried after written informed consent was obtained from the participants. All potential participants who declined to participate or otherwise did not participate were not disadvantaged in any way by not participating in the study.

### Subjects

Twenty-eight patients and twenty-eight healthy controls were recruited for the study. Data of four healthy controls and four patients were excluded from the final analysis because of low trial numbers due to artifacts (<50 artifact-free trials in the deviant conditions). The final sample comprised twenty-four patients with schizophrenia and twenty-four healthy controls. Healthy control participants were matched individually to schizophrenia patients by gender, age (+/−5 years), and years of education (+/−3 years), resulting in 24 matched pairs. All participants were right-handed with the exception of three left-handed patients and two left-handed healthy controls and had normal or corrected-to-normal vision. Participants did not receive payment for their participation. Data from the control group were published in part in Stefanics et al. [25].

Patients were recruited from the Department of Psychiatry and Psychotherapy of the Semmelweis University, Budapest, Hungary, from both the inpatient and outpatient units. Patients met the criteria for schizophrenia based on the Structured Clinical Interview for DSM-IV (Diagnostic and Statistical Manual of Mental Disorders, Fourth Edition [27]) Axis I Disorders. A trained psychiatrist or psychologist evaluated psychiatric symptoms on the Positive and Negative Syndrome Scale (PANSS) [28]. At the time of testing all patients were on antipsychotic medication (**Table 1**). To recruit a homogenous patient sample, besides outpatients, inpatients only before discharge were recruited into the study, which is reflected in the low overall PANSS scores (**Table 2**).

**Table 1:** Antipsychotic medications.

Antipsychotic Medication	Number of Patients (n)	Mean Daily Dose (SD) in milligrams
Amisulpride	5	700 (264)
Aripiprazole	3	20,0 (8.7)
Clozapine	6	198 (129)
Haloperidol	1	3,0 (0)
Olanzapine	3	15 (5)
Quetiapine	4	600 (294)
Risperidone	9	5.2 (2.5)
Zuclopenthixol	1	67,0 (0,0)*

* Weekly dose.

**Table 2:** Basic demographic and descriptive characteristics of the two study groups.

	Patients with	Healthy Control
	Schizophrenia *(n = 24)*	*Subjects (n = 24)*
Gender (Male/Female)	13/11	13/11
Age (years)	34.2 (10.3)	33.2 (9.8)
Education (years)	13.9 (10.1)	15.0 (2.6)
Handedness (right/left)	21/3	22/2
Symptom Checklist 90 (Global Severity Index)	98.6 (66.6)	22.9 (23.5)
Schizophrenia Subtypes: Paranoid/Catatonic/Disorganized/Undifferentiated	13/2/6/3	N/A
Inpatients/Outpatients	9/15	N/A
Duration of illness (years)	9.7 (7)	N/A
PANSS total	59.4 (21.6)	N/A
PANSS positive	14.5 (6.0)	N/A
PANSS negative	15.1 (7.5)	N/A
Antipsychotic medication (Atypical/Typical)	23/1	N/A
Chlorpromazine equivalent (mg)	601.9 (445.5)	N/A

Patients and controls were excluded if they had any other DSM-IV Axis I disorder, any central nervous system disease, mental retardation, history of head injury with loss of consciousness for more than 1 h, and alcohol or drug abuse. In case of controls, a short interview was performed by a trained physician for screening. According to the Derogatis criteria for ‘caseness’ (i.e.: high risk for a psychiatric disorder), a global severity index of >114 on the SCL-90 was an additional exclusion criteria for controls [29]. (114 was the T score of 63 on a Hungarian population sample [30]). No subjects from the Stefanics et al. 2012 investigation [25] were excluded from the control group based on these criteria.

The following clinical and emotion recognition measures were obtained from all participants before EEG recordings: the SCL-90, a 90-item Symptom Checklist assessing general dimensions of psychopathology, and the Ekman-60 Test (Facial Expressions of Emotion – Stimuli and Tests, FEEST) [31], a computerized emotion recognition test of 60 trials, where participants have to indicate what facial expression from the six basic emotions they think is displayed in the face by using the computer’s mouse to point and click on the appropriate emotion label on the screen.

Demographic information for both groups and clinical characteristics of the schizophrenia group are presented in **Table 2**.

### Stimuli and Procedure

**Figure 1:**
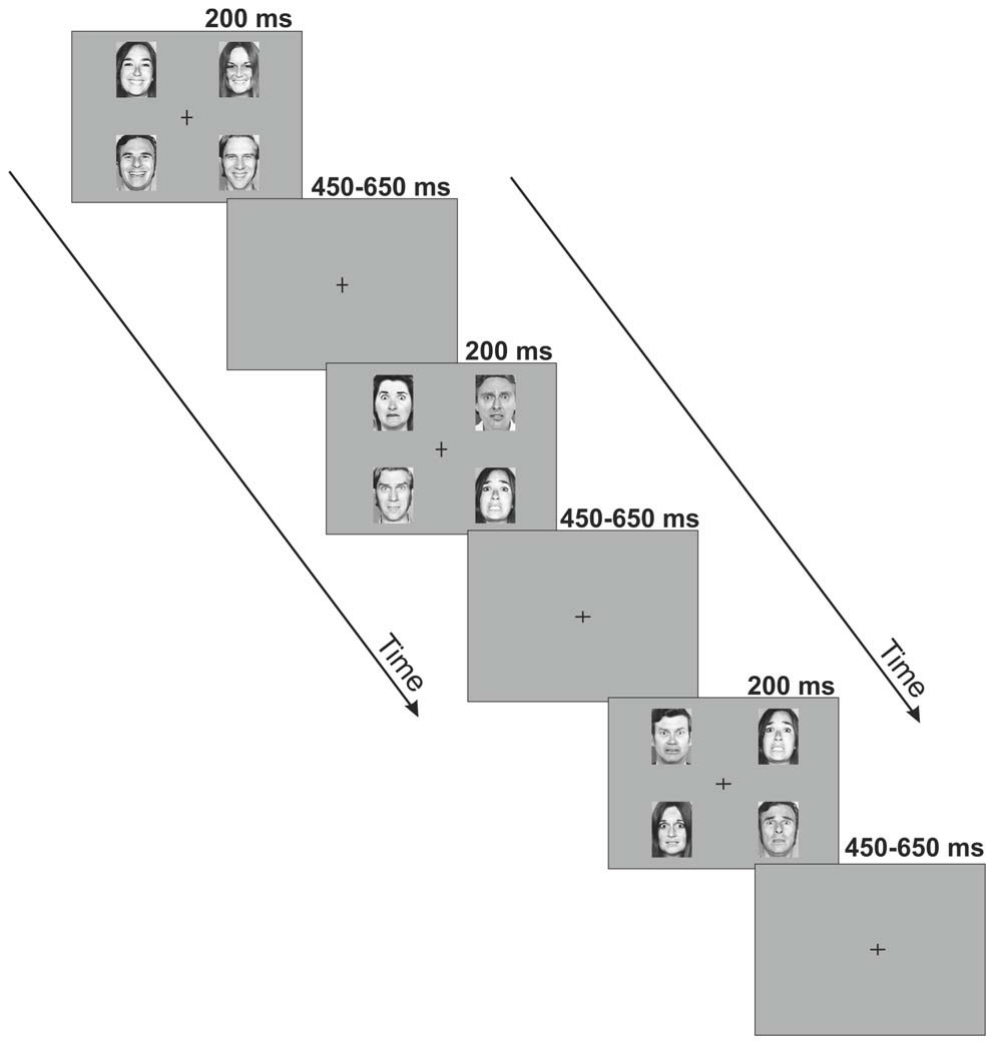
Stimuli and paradigm. Schematic illustration of the pattern of emotional stimuli used in the experiment. Four individual photographs displaying the same facial affect were presented on each screen for 200-stimulus interval randomly varying between 450–650 ms during which occasionally the vertical and horizontal lines of the fixation cross changed. The subjects’ task was a speeded button-press to the changes of the cross.

Visual stimuli were presented on a computer monitor. Stimulus presentation was designed in a manner to facilitate the forming of memory traces to emotions rather than to individual faces. To this end, black and white photographs of 5 female and 5 male faces were used as stimuli, taken from the Pictures of Facial Affect set [31] which is a standard set of stimuli in the field of facial emotion research, and has been used in many studies in the past decades. On each screen, 4 images of faces expressing the same emotion, specifically, images of 2 males and 2 females expressing the same facial emotion were presented in the upper-left, upper-right, lower-left and lower-right quadrants of the monitor. There are two advantages of this stimulus arrangement. First, faces presented outside the center of the visual field enable studying mismatch responses to deviants without attentional confounds. Second, using four different faces on each stimulus panel likely prevents local adaptation effects to contribute to possible deviance effects. In the center of the monitor a black fixation cross was presented. Pictures appeared on a dark-grey background at a viewing distance of 0.5 m. **Figure 1** illustrates the stimuli used in the experiment. Each face was subtended by 5.6° visual angle horizontally and 7.7° vertically. The distance of the inner corner of the pictures from the fixation cross was 4.4 visual angle horizontally and 3.8 visual angle vertically The presentation order of the individual pictures was randomized with the restriction that a picture of the same person was not presented on subsequent stimulus displays. Stimulus duration was 200 ms. The stimulus onset asynchrony (SOA) was randomized between 650–850 ms. In two experimental blocks fearful facial emotions were presented as frequent standards and happy facial emotions were presented as rare deviants (standard *P* = 0.9, deviant *P* = 0.1). In the remaining two blocks the standard and deviant emotions were swapped. The order of the four blocks was randomized across participants. A total of 100 deviant and 900 standard stimuli were presented for each emotion. The task of the subjects was a feature detection task entirely unrelated to the change in the facial expressions: they had to respond with a speeded button-press to the unpredictable changes in the length of either the horizontal or vertical lines of a black fixation cross presented in the center of the visual field. From time to time, the cross became either wider or longer, with a mean frequency of 11 changes per minute (SD = 3).

### EEG Recording and Preprocessing

EEG was recorded from DC with a low-pass filter at 100 Hz using a high-density 128-channel BioSemi ActiveTwo amplifier [32]. The electrode caps had an equidistant-layout and covered the whole head. EOG electrodes to monitor eye movements were placed below the left and above the right external canthi. Data were digitized at 24 bit resolution and a sampling rate of 512 Hz. Built-in and self-developed functions as well as the freeware EEGLAB toolbox [33] in the Matlab (MathWorks, Natick, MA) development environment were used for subsequent off-line data analyses. EEG was re-referenced to the common average potential and filtered off-line between 0.1 and 30 Hz using zero-phase shiftforward and reverse IIR Butterworth filter.

600 ms activity following the onset of the stimuli were extracted from the continuous EEG. The pre-stimulus period was 100 ms, which was used as baseline for the ERP generation. For both facial emotions, epochs were averaged separately for standards and deviants. Trials occurring within an 800 ms interval after a target event (i.e., change in the fixation cross) were automatically excluded from the analysis. To avoid potential artifacts, epochs with values exceeding ±120 µV on any EEG or EOG channel were rejected from the analysis. The mean number (and SD) of accepted trials for fearful and happy deviants and fearful and happy standards were 77 (8.4), 77 (6.9), 567 (62.3) and 561 (66.8) in the control group, and 75 (9.6), 86 (11.5), 533 (70.4) and 536 (70.8) in the schizophrenia group, respectively.

### Data Analysis

#### Generation of difference waveforms

**Figure 2:**
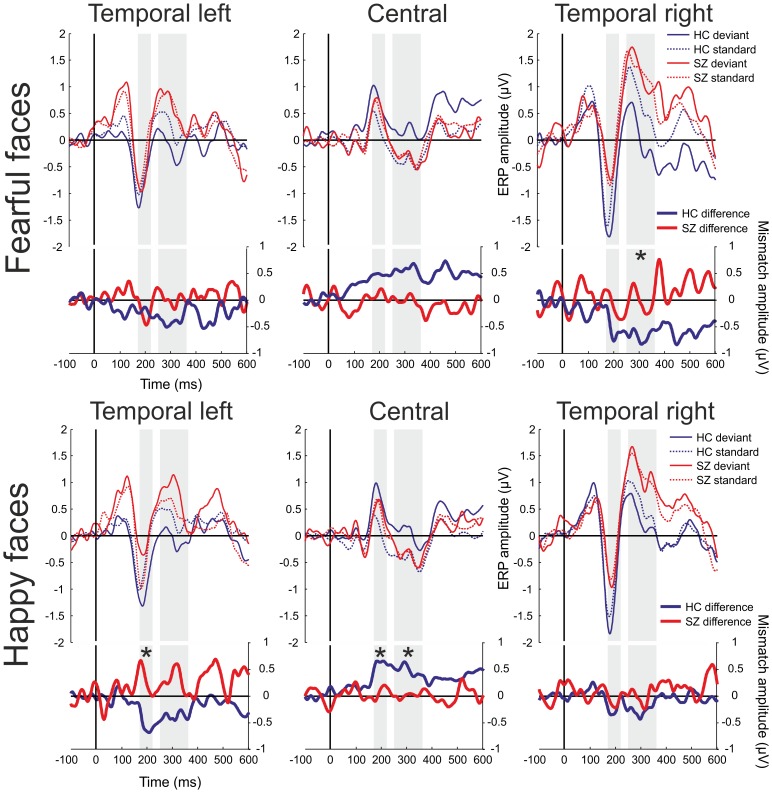
Event-related potentials and mismatch waveforms by region. HC = Healthy Controls, SZ = Patients with Schizophrenia. Upper panel: ERPs for fearful faces; lower panel: ERPs for happy faces. Shaded intervals indicate time windows of amplitude measurements. Only those ROIs were used for between-group comparison where the mismatch waveform in at least one of the study groups differed significantly from zero after correction for multiple testing. Asterisks mark time windows where significantly larger mismatch responses were found in the healthy control group compared to the patients.

**Figure 3:**
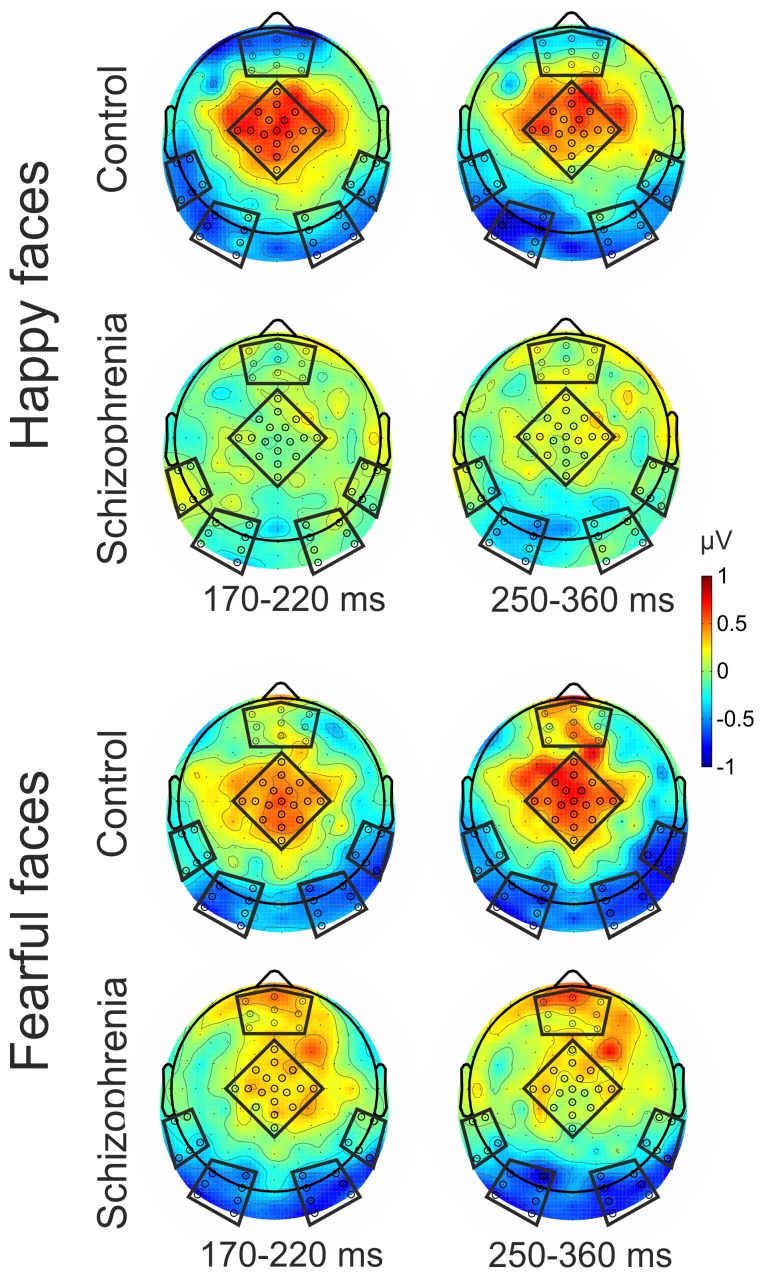
Scalp topography of the mismatch responses. Electrode clusters selected for analyses are marked with black dots in black frames (Region of Interests: ROIs).

Difference waveforms (mismatch responses) were created by subtracting ERPs to standards from the ERPs to deviants, separately for the two emotions **(** **Figure 2** **)**. In half of the blocks the roles of deviants and standards were reversed, responses to standard fearful faces were subtracted from responses to deviant fearful faces, and responses to standard happy faces were subtracted from responses to deviant happy faces. The only difference between standard and deviant emotions was the frequency of presentation in the given block. Since exactly the same pictures were used as deviants and standards, responses to physically identical stimuli were subtracted to calculate mismatch responses. Six Regions of Interest (ROIs) were formed (pre-frontal, central, temporal left, temporal right, occipital left and occipital right) according to previous visual mismatch studies [25], [34] **(** **Figure 3** **)**. Mean ERP responses were calculated by averaging across electrodes within ROIs. (Electrode clusters selected for analyses are marked with black dots in black frames in **Figure 3**).

The pre-stimulus baseline periods were compared between study groups in all ROIs and did not differ significantly after Hochberg correction [35] for multiple comparisons (p>0.5).

#### Study group comparison

Time windows of 170–220 ms and 250–360 ms were selected for analyses based on results from the same control population [25]. These time windows correspond well to those used in similar paradigms [4], [5], [36]. The early (170–220 ms) time window is thought to reflect activity related to the processing of emotions by the fusiform gyrus and the superior temporal areas [36], whereas the mismatch response in the later time window (250–360 ms) is thought to correspond with frontal generators [5]. The mean of the difference waveforms were calculated within these intervals and served as dependent variables in the main analysis. Group differences were characterized by Cohen’s *d*. For Cohen’s *d* an effect size of 0.2 to 0.3 is considered as “small” effect, around 0.5 a “medium” effect and >0.8, a “large” effect [37]. Difference between study groups was investigated by ANOVA with mismatch response amplitude as dependent and study group as independent variable. Only those ROIs were used for comparison where the mismatch waveform in at least one of the study groups differed significantly (*t*-test, *P*<0.05) from zero after Hochberg correction for multiple testing [35] across all ROIs. In other words, those ROIs were selected for study group comparison where the deviant and the standard waveforms differed significantly (i.e: the difference waveform represents a statistically validated mismatch signal). The ANOVA was done separately for the two emotions and two time windows. The *p*-values for the between-group comparison were also corrected for multiple comparisons (Hochberg correction) in each time window separately.

The rationale of this analysis strategy was twofold. First, a between-group difference is hardly explicable if no mismatch signal was found in any of the study groups. Second, by decreasing the number of the between group comparisons we can reduce the likelihood of Type II errors which may occur due to the adjustment for multiple comparisons.

#### Correlation with behavioral indices

In addition to investigating group differences, we also examined whether difference waveforms in the aforespecified time windows and regions exhibited any significant association with behavioral indices. Due to the non-normal distribution of the behavioral variables, Spearman rank correlation was used for these calculations.

#### Source Localization

The source activations for different conditions were compared using standardized low-resolution brain electromagnetic tomography (sLORETA, [38]–[40]). This method computes the cortical three-dimensional distribution of current source density of scalp-recorded electroencephalography (EEG). It provides a standardized discrete, three-dimensional distributed, linear, minimum norm inverse solution to the inverse problem of location of cerebral sources. The method uses the MNI152 template [41], with the three-dimensional solution space restricted to cortical gray matter, as determined by the probabilistic Talairach atlas [42]. The intracerebral volume is segmented into 6239 voxels with a 5 mm spatial resolution. Accordingly, sLORETA images reflect the standardized electric activity at each voxel in neuroanatomic Montreal Neurological Institute (MNI) space as the exact magnitude of the estimated current density. It has been confirmed that this method achieves zero localization error in noise-free stimulations [38]. Brodmann areas are also reported using MNI space, with correction to Talairach space [43].

## Results

Event-related potentials and difference potentials are shown in **Figure 2**, while **Figure 3** displays the scalp distributions of difference potentials. Deviant minus standard difference waveforms were negative in the occipital (**Figure 3** and Supporting Information: **Figure S1**) and temporal regions and positive in the central region (**Figures 2** **–** **3**).

### Behavioral Results

Reaction times and hit rates for the occasional changes in the fixation cross as well as false alarm rates were compared between study groups. A *t*-test of reaction times showed no significant differences between the blocks. Mean reaction times were 358 ms (SD = 117) for controls and 338 ms (SD = 160) for patients (*t* = 0.49, *P = *0.62. Hit rate was above 94% for both study groups; nonetheless, controls (97.8% SD = 1.8) significantly outperformed patients (94.2% SD = 3.9) (Kruskal-Wallis Test: *Chi^2^* = 8.5, *P*<0.005). False alarm rate was calculated as the ratio of button presses which were not preceded by a cross-flip in a 2000 ms interval before the event to the actual number of cross-flips. Mean false alarm rates were 1.1% (SD = 1.0) for controls and 2.5% (SD = 2.6) for patients respectively (Kruskal-Wallis Test: *Chi^2^* = 4.1, *P*<0.05). High hit rates and low false alarm rates even in the patient group made it unlikely that patients failed to direct their attention to the task.

### Mismatch Responses for Fearful Faces

In the control group a significant mismatch response was detected in the 250–360 ms time window over the left and right occipital and right temporal regions. No significant mismatch response was detected in the schizophrenia group in any of the ROIs for fear condition. Significant group difference (*F*(1,46) = 6.6, n = 48, *P = *0.01) was found in the 250–360 ms time window over the right temporal region (**Table 3**). This difference was 0.75 in terms of the effect size measure Cohen’s d (SD). The difference between study groups did not reach significance over the occipital regions (*P*>0.1).

**Table 3:** Between group differences in mismatch responses by region and emotion stimulus.

			Deviant vs. Standard				Between Group Difference in Mismatch Signal
			Control Group		Schizophrenia Group		
Condition	Time Window	Region of Interest (ROI)	LSMean(SE)	p value	LSMean(SE)	p value	Effect size (Cohen’s D)
	170–220 ms	Pre-Frontal	0.10 (0.25)	–	0.41 (0.25)	–	0.26
		Central	0.48 (0.20)	0.024	0.10 (0.20)	–	0.38
		Temporal Left	−0.21 (0.15)	–	−0.28 (0.15)	0.064	0.09
		Temporal Right	−0.54 (0.21)	0.014	−0.08 (0.21)	–	0.45
		Occipital Left	−0.52 (0.24)	0.035	−0.54 (0.24)	0.027	0.02
Fear		Occipital Right	−0.55 (0.23)	0.018	−0.39 (0.23)	0.087	0.15
	250–360 ms	Pre-Frontal	0.25 (0.26)	–	0.28 (0.26)	–	0.02
		Central	0.57 (0.25)	0.024	−0.06 (0.25)	–	0.53
		Temporal Left	−0.38 (0.20)	0.056	0.01 (0.20)	–	0.42
		**Temporal Right**	**−0.67 (0.18)**	**<.001** *	**−0.02 (0.18)**	**–**	**0.75** a
		Occipital Left	**−0.61 (0.18)**	**0.001** *	−0.41 (0.18)	0.027	0.23
		Occipital Right	**−0.60 (0.18)**	**0.002** *	−0.43 (0.18)	0.021	0.19
	170–220 ms	Pre-Frontal	−0.31 (0.23)	–	0.09 (0.23)	–	0.36
		**Central**	**0.61 (0.14)**	**<.001** *	**0.07 (0.14)**	**–**	**0.81** a
		**Temporal Left**	**−0.57 (0.20)**	**0.007** *	**0.32 (0.20)**	**–**	**0.89** a
		Temporal Right	−0.24 (0.20)	–	−0.12 (0.20)	–	0.12
		Occipital Left	−0.51 (0.27)	0.064	−0.12 (0.27)	–	0.30
Happy		Occipital Right	−0.38 (0.27)	–	−0.20 (0.27)	–	0.14
	250–360 ms	Pre-Frontal	−0.06 (0.21)	–	0.19 (0.21)	–	0.25
		**Central**	**0.45 (0.13)**	**0.002** *	**0.00 (0.13)**	**–**	**0.68** a
		Temporal Left	−0.38 (0.19)	0.053	0.30 (0.19)	–	0.73
		Temporal Right	−0.26 (0.19)	–	0.01 (0.19)	–	0.29
		Occipital Left	**−0.70 (0.21)**	**0.001** *	−0.18 (0.21)	–	0.53
		Occipital Right	−0.43 (0.22)	0.054	−0.10 (0.22)	–	0.31

* p<0.05 significant difference in ERPs to deviant and standard stimuli (significant mismatch signal) after Hochberg correction for multiple comparisons.

a p<0.05 significant difference in mismatch signal between groups.

Antipsychotic medication dose and symptom severity (PANSS total, positive and negative scores) did not correlate with the mismatch signals in these time windows over the above ROIs (*P*>0.5).

### Mismatch Responses for Happy Faces

In the control group a significant mismatch signal was detected over the central and the left temporal region in the 170–220 ms time window, while no mismatch was detected in any of the ROIs in the schizophrenia group (**Table 3**). A significantly larger mismatch response was observed in the control group compared to the patient group over the central (*F*(1;46) = 7.9, n = 48, *P = *0.007) and the temporal left (*F*(1;46) = 9.1, n = 48, *P* = 0.003) regions. These difference were 0.81 and 0,89 SD respectively.

In the control group in the 250–360 ms time window a significant mismatch signal was detected over the central and occipital ROIs, and again, no significant mismatch was detected in the schizophrenia group in any of the ROIs (**Table 3**). The difference between the groups was significant over the central region (*F*(1;46) = 5.5, n = 48, P = 0.02, Cohen’s D = 0.68).

Antipsychotic medication dose and symptom severity (PANSS total, positive and negative scores) did not affect the mismatch signals in these time windows over the above ROIs (*P*>0.1).

### Emotion Recognition and its Association with the Mismatch Responses

Behavioral performance on the emotion recognition task as indexed by the Facial Expressions of Emotion – Stimuli and Tests (FEEST) significantly differed between the study groups (Kruskal-Wallis Test: *Chi^2^* = 6.99, n = 45, *P = *0.008). The mean correct recognition scores (Control group = 85.9%, (SD = 7.5), Schizophrenic group = 79.0%, (SD = 9.3)) indicated a deficit in emotion recognition in the patient group. The effects size was 0.82 SD. Due to technical difficulties three healthy control subjects’ emotion recognition scores were not obtained thus only n = 21 control participants’ data were entered in group comparison.

**Figure 4:**
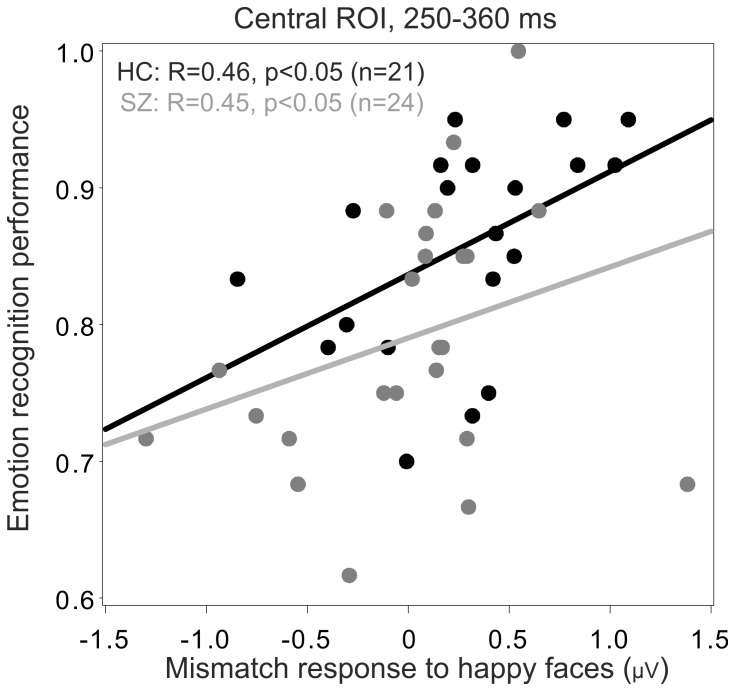
Association between recognition accuracy and mismatch responses. Subjects with Schizophrenia: Grey, Control Subjects: Black; ROI = Region of Interest. More positive mismatch signals were associated with higher recognition rates in this region.

Mismatch response to happy condition in the central ROI correlated significantly with overall emotion recognition (Spearman *R* = 0.49, n = 45, *P*<0.001), after correction for multiple testing. This correlation was significant in both study groups (Controls: *R* = 0.46, n = 21, *P*<0.05; Patients: *R* = 0.45, n = 24, *P*<0.04). All correlations were controlled for age and gender. More positive mismatch signals were associated with higher recognition rates in this region (**Figure 4**). No significant association between mismatch response and emotion recognition was found in the other ROIs.

### Association between Emotion Recognition Performance, Symptom Severity, and Antipsychotic Medication Dose in the Schizophrenia Group

Symptom severity (PANSS total, positive and negative scores) and antipsychotic medication did not affect emotion recognition performance (Spearman rank correlation, *P*>0.4).

### Source Localization of the Mismatch Responses by sLORETA

The source activations underlying the scalp ERP waveforms were calculated for each subject using a statistical nonparametric mapping method based on the sLORETA toolbox. First, voxel-by-voxel comparisons were made between standard and deviant stimuli within the groups separately for the 2 emotion conditions, and thereafter between the mismatch signals of the two study groups by independent *t*-test (Control Group Deviant minus Control Group Standard vs. Schizophrenia Group Deviant minus Schizophrenia Group Standard). Statistical significance was assessed with a nonparametric randomization test (n = 5000) that corrects for multiple comparisons [44]. Source locations were estimated for the 170–220 and 250–360 ms time windows.

**Figure 5:**
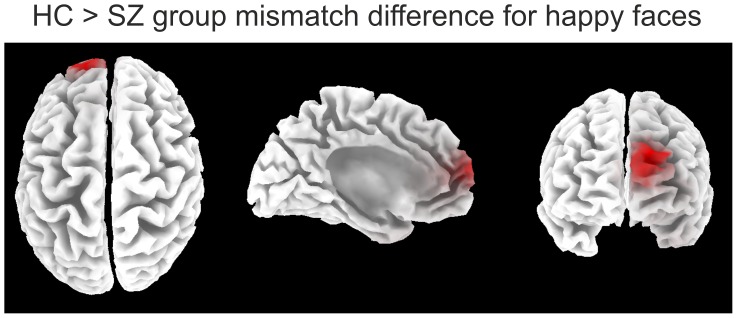
Results of the source localization for the happy condition. Red color indicates significant group differences in mismatch generation to the happy condition in the 250–360 ms time window. (HC = Healthy Controls, SZ = Patients with Schizophrenia).

In the Schizophrenia group, no difference was observed between standard and deviant stimuli in any of the time windows for either condition. In the Control group significant differences were found between standard and deviant stimuli in the 170–220 ms time window at (*P*<0.1) level and in the 250–360 ms period at (*P*<0.05) level for both emotion conditions in frontal regions (**Table 4**). Group comparison revealed a significantly attenuated activity in the 250–360 ms time window for both emotion conditions (*P*<0.05 for happy condition, and *P*<0.1 for fear condition) in frontal regions in the Schizophrenia group. Results are summarized in **Table 4** and **Figure 5**.

**Table 4:** Source Localization of the Mismatch Signals.

	Study Group(s)	Condition(s)	Time window	Areas^1^	Brodmann	MNI coordinates at t_max_		
					Areas	*X*	*Y*	*Z*
			170–220 ms	Middle Frontal Gyrus(*)	11	−45	45	−15
				Superior Frontal Gyrus(*)	8	25	25	55
			250–360 ms	Middle Frontal Gyrus*	6, 8, 10, 11, 47	−40	55	−5
		Happy Deviant		Superior Frontal Gyrus*	6, 8, 10, 11	30	25	55
*Within*	Control Group	vs.		Inferior Frontal Gyrus*	10, 47	−40	55	5
*Group*		Happy Standard		Sub-Gyral*	8, 10	−40	45	0
*Comparison*				Cingulate Gyrus*	6, 24, 32	20	5	50
				Medial Frontal Gyrus(*)	6, 8, 10, 32	15	10	50
				Anterior Cingulate(*)	32	−20	45	10
	Schizophrenia Group	Happy Deviant vs.	170–220 ms	No Significant Difference	–	–	–	–
		Happy Standard	250–360 ms	No Significant Difference	–	–	–	–
			***170–220*** ***ms***	***No Significant Difference***	***–***	***–***	***–***	***–***
***Between***	***Control Group***	***Happy***	***250–360*** ***ms***	***Superior Frontal Gyrus*** *	***9, 10***	***−15***	***60***	***25***
***Group***	***vs.***	***Mismatch***		***Middle Frontal Gyrus*** *	***10***	***−20***	***60***	***25***
***Comparison***	***Schizophrenia Group***			***Medial Frontal Gyrus*** *	***9, 10***	***−5***	***55***	***20***
				***Anterior Cingulate*** *	***32***	***−5***	***45***	***15***
		Fear Deviant	170–220 ms	Superior Frontal Gyrus(*)	8, 9	30	45	40
*Within*	Control Group	vs.		Middle Frontal Gyrus(*)	8, 9	25	45	40
*Group*		Fear Standard	250–360 ms	Superior Frontal Gyrus*	6	−15	20	65
*Comparison*	Schizophrenia Group	Fear Deviant vs.	170–220 ms	No Significant Difference	–	–	–	–
		Fear Standard	250–360 ms	No Significant Difference	–	–	–	–
***Between***	***Control Group***	***Fear***	***170–220*** ***ms***	***No Significant Difference***	***–***	***–***	***–***	***–***
***Group***	***vs.***	***Mismatch***	***250–360*** ***ms***	***Superior Frontal Gyrus(*** * ***)***	***6***	***−15***	***20***	***65***
***Comparison***	***Schizophrenia Group***							

* p<0.05, two-tailed, ^(^*^)^ p<0.1, two-tailed.

1 Areas listed by t_max_ in decreasing order.

## Discussion

To our knowledge the current study is the first to compare visual mismatch responses, an index of automatic predictive mechanisms, to unattended facial expressions between patients with schizophrenia and controls. Non-conscious expectations were induced by frequent repetitions of unattended faces (standard) expressing a particular emotion, and this expectation was violated by faces expressing another emotion (deviant). ERPs to physically identical deviant and standard stimuli were compared to control for possible effects for differences in low-level physical features. Although this method does not control for possible refractoriness effects per se as equiprobable paradigms do [45], [46], we interpret the observed mismatch activity as prediction error responses to ‘unexpected’ emotions, since in the current study pictures of several male and female models were used to avoid the possibility of low-level adaptation to features of a particular face. Thus predictive memory representations were formed for emotions, rather than to individual faces.

### Diminished Visual Mismatch Responses in the Schizophrenia Group and Differences with Controls

In the schizophrenia group, a tendency for mismatch responses was detected over the occipital regions for the fear condition in the 170–220 ms and 250–360 ms time windows **(** **Table 3**, Supporting Information: **Figure S1)**. However, after correction for multiple testing mismatch responses in the patient group did not reach significance for any of the emotional conditions. These findings are in line with the results of Urban et. al., who also reported decreased visual mismatch responses in a schizophrenic group in a motion-direction oddball paradigm [19].

In the control group mismatch responses were detected for fearful faces in the left and right occipital and in the right temporal regions in the 250–360 ms time window. Between study groups, the difference was significant over the right temporal region for fearful faces (**Figure 2**). In the control group, for the happy condition, mismatch responses were detected in the 170–220 ms time window in the left temporal and central regions, and in the 250–360 ms time window in the left occipital and central regions, and again no such effects emerged in the schizophrenic group. Group differences were significant and showed large effects sizes in the 170–220 ms time window in the left temporal and central regions and in the 250–360 ms time window in the central region for happy faces (**Table 3** **and** **Figure 2**).

Medium-to-high effect sizes were also detected for both time windows for both conditions, but they did not reach significance due to the relatively small sample size. Furthermore, significant group differences were excluded where the difference between deviant and standard stimulus did not reach significance in any of the study groups (e.g. Left Temporal ROI late time window, happy stimulus). The largest effect sizes between study groups were in the 0.75–0.89 range (**Table 3**), which fall well within the 95% confidence limits determined by a meta-analysis of auditory MMN studies in schizophrenia (mean effect size = 0.99, 95% CI:0.79–1.29) [7]. This indicates that the magnitude of the deficit in mismatch generation in the visual modality is comparable to that detected in auditory modality. Our results showed that the visual (emotion processing) system was capable of detecting the difference between frequent (standard) and rare (deviant) stimuli in healthy participants, while the same detection process was impaired in patients with schizophrenia. Alternatively, it is conceivable that the build-up of the expectation for a reappearing (repeating) emotion might have failed in schizophrenia patients, thereby preventing the elicitation of a mismatch response. In either case, our results demonstrate that impairment of emotion processing in schizophrenia is present already at the automatic unconscious level. The fact that the severity of psychotic symptoms did not influence the mismatch signals in the specified regions and time windows support the notion that this is rather a trait- than a state-like deficit in schizophrenia.

In the auditory modality diminished MMN in schizophrenic patients has been attributed to N-methyl-D-aspartate (NMDA) receptor-mediated glutamate dysfunction [47], [48]. NMDA antagonists have been shown to diminish MMN amplitude in animal models [49]. It is possible that a similar receptor mechanism may underlie the generation of visual MMN and that visual mismatch deficits in schizophrenia might be caused by altered modulation of NMDA receptor-related synaptic plasticity [18], [50]. However, further studies are required to evaluate this possibility.

### Relationship between Mismatch Responses and Emotion Recognition

Mismatch responses with positive polarity were observed in central regions for both emotional conditions. Previous studies applying oddball paradigms have observed this positive response mainly in central and anterior regions [8], [51]. It has been proposed that responses of fusiform sources to face stimuli in scalp EEG recordings usually manifest as positivities at the vertex [52], [53]. The fusiform gyrus is a face-selective area [54], and might have contributed to the processing of facial emotions in our experiment.

One of the key findings of the present study is that mismatch responses showed an association with emotion recognition performance. Mismatch response amplitude for happy faces positively correlated with overall emotion recognition and was significantly more positive in the control group relative to the patient group in the central region (250–360 ms time window).

To our knowledge this is the first study to demonstrate a relationship between mismatch signals and emotion recognition performance. Earlier studies showed that auditory MMN impairments can be linked to cognitive [55] and everyday functioning [56]. Light and Braff [56] suggested that MMN deficits represent a core neurophysiological dysfunction, which is linked to global impairments in everyday functioning in schizophrenia patients. They found that deficits in automatic preattentive information processing, as measured by MMN, strongly correlated with global functioning in subjects with schizophrenia, although they did not find a relationship between symptom severity, laboratory-based measures of functional capacity (UPSA), and mismatch amplitudes. Social functioning is also strongly correlated with social cognition and facial affect recognition [57], [58]. These findings, taken together with our results support the notion that emotion recognition deficits might be mediators between automatic preattentive information processing deficits and everyday life functioning impairments in schizophrenia. A future study applying a visual mismatch paradigm to test facial emotion processing should also include measures of facial affect recognition and social functioning to confirm this notion.

### Source Localization

The estimated sources of the mismatch responses for both emotional conditions were localized in prefrontal regions. This is in line with prior results [5] showing that sources of visual MMN to emotional faces in healthy subjects were located in frontal and temporal cortices. These regions play a key role in the formation and updating of visual predictions [16], [59]. A recent study [60] reported decreased source activity to emotional faces in frontal regions in patients with schizophrenia compared to controls. Taken together with our findings, these results also corroborate the notion that impaired functioning of frontal-prefrontal brain regions might be an underlying cause of deficits in emotion recognition in schizophrenia. The finding that sLOTERA localized the source of the MM signal in the frontal region and yielded a significant between group difference while the effect size did not reach statistical significance for this ROI on the scalp maybe due to the fact the EEG signal dampens, and undergoes spatial blurring while transmitted to broad regions of the scalp [61]. However, with the dense electrode array we used, sLORETA captures this spatially distributed information since it calculates the sources of the EEG signal by solving the inverse problem based on all electrodes.

Contrary to our expectations and previous results [5], neural generators were not identified in the temporal gyrus (e.g. in the Fusiform Face Area). A possible explanation is that simultaneously active sources can only be separated by sLORETA if their fields are distinct enough and of similar strength. In the context of a strong or superficial source, weak or deep sources remain invisible for this method, and nearby sources of similar orientation tend not to be separated but interpreted as one source located roughly in between [62]. Future studies with better resolution will be needed to clarify this issue.

### Limitations and Future Directions

A main limitation of our study is that all patients had been receiving psychotropic medication at the time of testing. However, no correlation was found between antipsychotic dose and mismatch signals, which is in line with previous findings, namely that D2 or 5 HT2 antagonist antipsychotics such as Clozapine and Olanzapine do not influence MMN amplitude [63], [64]. Another limitation is that the investigation was cross-sectional and the average PANSS scores were low, indicating a chronic-stable mental state [65], which may have limited our ability to find a correlation between symptom severity and mismatch signals. Further longitudinal studies are needed to clarify this association. Since our aim was to study emotion recognition, in the present study specific visual stimuli (i.e. emotional facial expressions) were used. Accordingly, further research should investigate the correlation between MMN to simpler visual stimuli and social cognition.

## Conclusions

Building up a predictive model based on the regularities of facial expressions around us and the comparison of any upcoming facial cue to this model can be the key to the unintentional recognition of others’ facial expressions in everyday life. Our findings support the notion that impaired generation of mismatch signals may indicate impairment in automatic processing of emotions in patients with schizophrenia, which leads to decreased emotion recognition and subsequently to a disability in social functioning.

## Supporting Information

Figure S1 **Event-related potentials and mismatch waveforms by region (HC = Healthy Controls, SZ = Patients with Schizophrenia).** Upper panel: ERPs for fearful faces; lower panel: ERPs for happy faces. Shaded intervals indicate time windows of amplitude measurements. Crosses mark time windows where deviant and standard waveforms differ significantly (i.e. significant mismatch waveform).
(EPS)
